# Uveo-Parotitic Paralysis. A Report of a Case and Some Account of Previous Instances

**Published:** 1926

**Authors:** George Parker

**Affiliations:** Consulting Physician to the Bristol General Hospital; Lecturer in Forensic Medicine and Toxicology in the University of Bristol


### The Bristol Medico-Chirurgical Journal

*"Scire est nescire, nisi id me
Scire alius sciret."*

SUMMER, 1926.

### UVEO-PAROTITIC PARALYSIS.

### A REPORT OF A CASE AND SOME ACCOUNT OF PREVIOUS INSTANCES.

BY

GEORGE PARKER, M.A., M.D.Cantab.

*Consulting Physician to the Bristol General Hospital; Lecturer in Forensic Medicine and Toxicology in the University of Bristol.*

---

As I have recently met with a mild attack of this rarely observed disorder, and as two other cases have appeared in Bristol, a short account of my patient and some discussion of the possible importance of the syndrome may be of interest.

The symptoms include an inflammation of the uveal tract in the eyes, a swelling of the parotid glands, facial paralysis, and often paresis of various muscles of the body, multiple neuritis, as well as an erythematous rash on the legs and trunk, with or without fever.

My patient, Miss S., aged 55, was attacked on January 24th, 1926, immediately after a journey to Clutton, with shivering and pains all over, especially in the head and chest. The mouth became dry and unpleasant, the eyesight misty, the eyes tender, and any attempt to read brought on headache. In the course of a fortnight she noticed a swelling on the face in front of the right ear with slight pain and tenderness. On February 8th she sent for me, thinking that the swelling might be due to mumps caught on the day of her journey. I found a moderate enlargement of the pre-auricular part of both parotids passing slightly below the jaw, but not extending into the neck behind the jaw. The right parotid was much larger than the left, and contained a hard nodule the size of a marble. She could yawn or gape without pain. There was no puffiness or redness, but the swellings were somewhat hard and showed little tenderness. The nasolabial fold on the left side of the face was much exaggerated, and it turned out that there was a definite facial paralysis on the right side, and possibly a less marked one on the left. She could feebly wrinkle the frontal muscles on both sides. The most striking feature, however, was the narrowing of the palpebral fissure on the left side with dropping of the eyebrow or pseudoptosis. The pupils of eyes were semi-dilated, did not accommodate to distance at all, and only feebly to light, but there was little or no ciliary congestion, no strabismus or optic neuritis.

It did not seem at all likely that mumps could be the cause of such a state of things as this. Even supposing that she came into contact with mumps on the 24th, the illness was immediate without time for incubation, and although facial paralysis has occasionally been seen in that disorder, the localised and almost painless swelling of the parotids with one or more hard nodules is unlike anything found in it.

Moreover, the parotid swellings did not entirely pass off for several weeks, and the slit-like palpebral fissure and the facial paralysis are still present to-day three months after the attack. A papular rash, too, has recently appeared on the forearms. In short, I was quickly satisfied that the case was one of uveo-parotitic paralysis. However, the attack was a very mild one. There was no neuritis or paræsthesia, no loss of knee, ankle or arm jerks, or of the vibration sense, no paresis of soft palate, no pyrexia (at least on the few occasions when I could take it), and no diphtheria bacilli in the throat. The heart, lungs and urine were normal. The vision quickly became good, and she remained at work. No one else in the house developed mumps.

As to her past history, she had always been a healthy, active woman, except for occasional attacks of muscular rheumatism yielding quickly to salicylates. In August—September, 1925, she had some gastro-intestinal trouble of obscure origin lasting for several weeks, but since then she had seemed very well, in spite of heavy work and the strain of nursing a sick friend. It may be worth mentioning that her dog was taken ill with distemper at the time of her illness.

If we now turn to the recorded cases, we find that the first account of the syndrome was given by Heerfordt at Copenhagen in 1909. He described three cases and unearthed two others by searching through the literature. Mackay, when bringing forward another in 1917, went through the *Index Medicus* up to that date, and I have continued the search through it up to now. From various sources I have found and collated sixteen cases, and there are five or ten others, several of them written in Danish or Norwegian, the accounts of which have not been accessible, making in all about twenty-five cases. Fredrik Berg thinks the number reported approaches forty, but the evidence from his paper is unsatisfactory. The condition, however, is probably more common than we are aware. In this country only six cases have been found hitherto. I have excluded some of the instances reported by Mackay, Dopter and others which seemed to be clearly mumps, and have not added the remarkable case of Leeksma, which he held to be mumps, since there had been possibilities of infection, though the long prodromal period, the iridocyclitis, and the facial paralysis coming before the parotitis, and finally the skin rash, when taken together point to something quite unlike that disorder.

I now add a tabular analysis of the sixteen cases. (See pp. 80 and 81.)

On looking over these reports the average age seems to be over 31 years. Males are fewer than females, which is the reverse of mumps. Heerfordt noted a premonitory period of two weeks to two months, and in several later cases there was malaise, vomiting or other trouble for from 5 to 30 days before the typical symptoms began. The order in which these latter appear shows great irregularity. Sometimes œdema or a rash on the limbs, sometimes parotitis, sometimes eye troubles, or the facial paralysis, may arise a week or more before the rest. The duration of the illness also varies greatly ; but although complete recovery is the rule, this is not usually reached for several months, and in one instance the parotitis lasted at least two years. The temperature may be normal throughout, or there may be slight continuous pyrexia, or a remittent fever with a weekly crisis for some months.

The parotid swellings are generally bilateral, painless, sometimes hard or nodular, often limited to the pre-auricular area or extending below the jaw, never suppurating, but lasting weeks or months.

The eye troubles include misty vision with more or less failure of sight, ciliary congestion, the pupils often irregular or dilated with little or no response to light or accommodation, vitreous opacity, keratitis punctata, occasionally increased tension and glaucoma, narrowing of the palpebral fissure, and pseudoptosis, occasionally optic neuritis or atrophy. There is generally a double asymmetrical facial paralysis of the peripheral type, and sometimes other cerebral or spinal nerves show signs of paralysis or multiple neuritis. Thus we may find dysphagia, deafness, paresis of legs or soft palate, pins and needles in hands and feet, intercostal and other pains, paræsthesiæ, numbness, and loss of taste. Absence of knee jerks, ankle jerks or arm jerks or of the vibration sense may occur. Skin eruptions are sometimes troublesome, such as an erythema on the legs and abdomen which may resemble erythema nodosum. Besides this there may be urticaria, or œdema in the ankles, legs and below the eyes, though usually no kidney or heart trouble appears. Very little success has attended the search for bacterial or other causes. Blood cultures, the Wassermann, von Pirquet, diphtheria, and other tests have generally been negative. Professor Carl Browning of Edinburgh made a complete serological and bacteriological search in Ramsay's case, including the urine, nose, blood and fæces, but could find nothing. Blood counts seem to be fairly normal. Fielding and

Viner noted a leucopenia in their case at first, which contrasts with the usual lymphocytosis in mumps. The spinal fluid here, however, showed fifteen cells, all lymphocytes per c.c., and MacBride found eight cells per c.c., including some large mononuclears. The Nonne-Apelt test was negative, or gave a slight haze.

We may perhaps consider the condition to be chiefly an infective multiple neuritis, showing certain lesions in other tissues, such as parotitis, uveitis, and skin troubles. Of course, there are forms of infective nerve disease where the incidence is confined to the nervous system, such as poliomyelitis, acute multiple neuritis, Landry's disease, if you will, and cerebro-spinal fever. In others there are lesions in various tissues. In E. lethargica we may have sore throat, skin rashes, and diarrhœa. In diphtheria, besides the widespread nerve affections, there is the throat trouble. In beriberi, if that is an infective neuritis, there are also eye lesions and œdema. The present disorder shows much resemblance to E. lethargica and to diphtheria, and in all three the loss of accommodation in the eye is a very frequent and early symptom. There is at present no evidence as to how the toxin enters the body, and whether it is distributed by the blood, perineural spaces or lymphatics. It has even been suggested that the syndrome is a deficiency disease or an endocrine one, but no evidence to speak of has been brought forward. A syphilitic or tuberculous cause has been much discussed by T. Mohr, Giessing, Rieth and others and on the other hand benefit from treatment by X-rays or thyroid gland has been claimed by Jackson and Ramsay.

Turning to the non-neural lesions, the parotitis presents many difficulties. Apart from mumps, this condition occurs, as is well known, after some acute fevers, pneumonia, abdominal operations, and where no food is given by the mouth, but these cases are definitely inflammatory and often go to suppuration. Again, during the war there were numerous chronic cases, not contagious and with no complications, but probably due to stomatitis. Finally, benign and malignant tumours in the parotid region and Mickulicz's disease need not detain us, for they present such a different picture.

The really important question is whether this syndrome is or is not an abnormal form of mumps. Before Heerfordt wrote such cases were ascribed to mumps,[1] and some writers, such as Leeksma, think so still. There is a large literature on the complications of mumps, and most of the symptoms discussed above have individually been found following it. Still, in none of our sixteen cases was any actual connection traced with mumps, nor did they give rise to fresh cases of it.

Antony Feiling has made a special study of both mumps[10] and this syndrome, and he concludes that they are absolutely distinct. Indeed, it is difficult to come to any other conclusion, for the order and the duration of the symptoms are so very different. So too are the characters and site of the swelling, the temperature and blood counts, the sex and age incidence. Thus mumps is most common in males (as even Hippocrates notices) from five to twenty-five years. It has a prodromal period of only twelve to thirty-six hours, the fever and parotitis last only from two to seven days, second attacks being almost unknown. Lymphocytosis is present in the blood generally, and almost always in the spinal fluid. If facial paralysis should occur it is unilateral, and follows after the parotitis. In fact, there is extremely little

| No. | Author. | Date. | Age. | Prodrome. | Duration of Final Stage. | Eyes. | Parotids. | Temperature. | Nerve Symptoms. | Skin, etc. |
|---|---|---|---|---|---|---|---|---|---|---|
| 1 | Heerfordt | 1909 | M. 11 | Gastritis, some weeks | 4½ months | Iridocyclitis, optic neuritis | Double swellings | Pyrexia for months | — | — |
| 2 | ,, | ,, | M. 14 | ,, | 2 months | Iridocyclitis | ,, | Slight. | Facial paralysis, dysphagia | — |
| 3 | ,, | ,, | M. 27 | ,, | ,, | ,, | ,, | ,, | Facial paralysis, dysphagia, paræsthesia | — |
| 4 | Daireaux and Pechin. | 1899 | M. 22 | — | — | Iritis, keratitis | ,, | — | — | — |
| 5 | Collomb | 1903 | M. 29 | Dyspepsia | — | Iridocyclitis | ,, | — | — | — |
| 6 | Brewerton | 1910 | M. 17 | Urticaria, 5 days | 4 months | Failure of vision, ciliary congestion, keratitis. | ,, (hard nodular) | Early stages were pyrexial | — | Erythema nodosum. |
| 7 | Kuhlefelt | 1916 | F. 21 | — | — | Iridocyclitis, neuro-retinitis | Double | Occasional pyrexia | — | — |
| 8 | Mackay | 1917 | F. 30 | Gastritis, 2 weeks | 2 months | Ciliary congestion keratitis punctata pupils dilated, no accommodation, no reaction to light | ,, | Pyrexia at first | — | — |
| 9 | T. Mohr | 1920 | F. 34 | — | — | Iridocyclitis and keratitis | ,, | — | — | Evidence of syphilis. |

| | | | | | | | | | | |
|---|---|---|---|---|---|---|---|---|---|---|
| 10 | Feiling and Viner | 1921 | F. 21 | Malaise, 1 month | 4 months | Ciliary congestion, pupils dilated, no reaction to light, no accommodation; glaucoma and paracentesis | ,, | — | Early facial paralysis, no knee jerks, no ankle jerks, paræsthesiæ | Erythema. |
| 11 | Maitland Ramsay | ,, | F. 31 | Œdema, 6 weeks | Some months | Ciliary congestion loss of vision, optic atrophy | ,, | — | Facial paralysis, paralysis of soft palate, intercostal neuralgia, colitis | Erythema. |
| 12 | MacBride | 1923 | F. 43 | Appendicectomy | 9 months | Iridocyclitis | ,, | Remittent, weekly fever | Paresis legs and arms, facial paralysis, dysphagia, deafness. | |
| 13 | Fredrik Berg | 1923 | F. 23 | — | — | Failure of vision, iridocyclitis and keratitis | ,, (late) | — | — | — |
| 14 | Critchley and Phillips | 1924 | F. 62 | — | 2 years | Pupils sluggish, ptosis, no accommodation | ,, (hard) | Slight pyrexia | Facial paralysis, loss of vibration sense | — |
| 15 | Coombs, Rogers and Bodman | 1926 | F. 61 | Gastritis, 18 months | 10 months | Dacryo-cystitis, pupils-irregular | ,, | Apyrexia in later stage | Paresis legs and arms, neuritis, paræsthesiæ, delirium. | Erythema. Mastitis. Death. |
| 16 | G. Parker | 1926 | F. 55 | — | 3 months | Pupils sluggish, no accommodation, ptosis, narrowed fissure | ,, (nodular) | — | Facial paralysis. | Papular rash. |

agreement. However, we are arguing from only the few cases recorded, and until the organism peculiar to mumps is isolated with certainty it is impossible to give an absolute decision.

The pathology of the eye symptoms is also obscure. There is clearly an inflammation of the uveal tract, but what is the toxin and how does it get access to this area ? Iridocyclitis occurs in typhoid, tuberculosis, syphilis, or during infections of the nasal cavities, of the alimentary tract and of the bladder. Much as it has been discussed very little is known with certainty about it. The combination with cycloplegia is much rarer. The skin rashes point to a general blood infection. Curiously they do not seem to occur in those cases where pyrexia is most marked.

In conclusion, the syndrome shows an acute polyneuritis with swelling of the parotids, inflammatory changes in the eyes and the skin, and in very severe cases, such as that mentioned by Dr. Coombs, it may go on to cerebral symptoms and death.

### Discussion.

Dr. Carey Coombs described the case reported in this issue by Drs. Beatrice Rogers and Hervey Bodman (who were unable to be present).

Dr. Phillips referred to the case reported by himself and Dr. Macdonald Critchley in 1924. The present was the third case occurring locally in recent times. His own case had not shown any features that distinguished it especially from the usual course.

Col. Lister mentioned the uveitis that sometimes accompanies beriberi, and suggested that the connection in these cases was perhaps similar.

REFERENCES.

Case 1. Heerfordt, *Archiv. f. Ophthal.*, 1909, p. 254.
Case 4. Daireaux, *Le Bulletin Medic.*, 1899, p. 227.
Pechin, *Rec. d'Ophthal.*, 1901, p. 336.
Case 5 Collomb, *Rev. med. de la Suisse romande*, 1903.
Case 6. Brewerton, *Trans. Ophthal. Soc.*, 1910, p. 148.
Case 7. Kuhlefelt, *Finska Lak-sallskis handlung*, 1916, p. 867.
Case 8. Mackay, *Trans. Ophthal. Soc.*, 1917, p. 208.
Case 9. T. Mohr, *Klin. Monatschr f. Augen.*, 1920, p. 694.
Case 10. Feiling & Viner, *Jour. of Neur. & Psychop.*, 1922, p. 353; *Quart. J. Med.*, 1915, p. 257.
Case 11. Maitland Ramsay, *Trans. Ophthal. Soc.*, 1921, p. 194.
Case 12. MacBride, *Jour. of Neur. & Psychop.*, 1923, p. 242.
Case 13. Fredrik Berg, *Hygiea*, 1923, pp. 402–418.
Case 14. Critchley & Phillips, *Lancet*, 1924, vol. 2 p. 906.

See also—

Dopter, *Gaz. des hôp.*, 1904, pp. 77-887.
Leeksma, *Nederl. Tijdschrift voor Geenesk*, 1916, and *Lancet*, 1917, i. 28.

Other papers on the subject not referred to in this article:—

S. Michaelsson, *Hygiea*, 1924, p. 344.
K. Lehmann, *Hospitalstid*, 1917, p. 1054.
S. Bang, *Ugeskrift f. Laeger*, 1918, p. 571.
B. N. Jackson, *Amer. Jour. Ophthal.*, 1925, p. 361.
— Lerner, *New York State Jour. Med.*, 1924, p. 297 597.
G. F. Bentzen, *Forhandl. i. det Oftal Selsk.*, 1917–18, p. 14.
H. Giessing, *Klin. Monatsch. f. Augen.*, 1918, p. 249.
S. Schou, *Klin. Monatsch. f. Augen.*, 1924, p. 281.
C. W. Cutler, *Trans. Amer. Ophthal. Soc.*, 1904, p. 390.
H. Thomsen, *Ugeskr. f. Laeger*, 1919, p. 39.

